# Side-by-side comparison of parent vs. technician-collected respiratory swabs in low-income, multilingual, urban communities in the United States

**DOI:** 10.1186/s12889-022-12523-3

**Published:** 2022-01-15

**Authors:** Sandra S. Chaves, Ju-Hyeong Park, Mila M. Prill, Brett Whitaker, Reena Park, Ginger L. Chew

**Affiliations:** 1 CDC, National Center for Immunization and Respiratory Diseases, Influenza Division, Atlanta, USA; 2 CDC, National Institute for Occupational Safety and Health, Respiratory Health Division, Morgantown, USA; 3 CDC, National Center for Immunization and Respiratory Diseases, Division of Viral Diseases, Atlanta, USA; 4 Marshall University Joan C. Edwards School of Medicine, Huntington, USA; 5 CDC, National Center for Environmental Health, Division of Environmental Health Science and Practice, Atlanta, USA

**Keywords:** Home-swabbing, Parental swabbing, Pathogen detection, Upper respiratory tract samples, Dry tube for specimen transport

## Abstract

**Background:**

Home-based swabbing has not been widely used. The objective of this analysis was to compare respiratory swabs collected by mothers of 7–12-year-olds living in low-income, multilingual communities in the United States with technician collected swabs.

**Methods:**

Retrospective data analysis of respiratory samples collected at home by mothers compared to technicians. Anterior nasal and throat specimens collected using flocked swabs were combined in dry tubes. Test was done using TaqMan array cards for viral and bacterial pathogens. Cycle threshold (Ct) values of ribonuclease P (RNP) gene were used to assess specimen quality. Ct < 40 was interpreted as a positive result. Concordance of pathogen yield from mother versus technician collected swabs were analyzed using Cohen’s Kappa coefficients. Correlation analysis, paired t-test, and Wilcoxon signed-rank test for paired samples were used for RNP Ct values.

**Results:**

We enrolled 36 households in Cincinnati (African American) and 44 (predominately Chinese or Latino) in Boston. In Cincinnati, eight of 32 (25%) mothers did not finish high school, and 11 (34%) had finished high school only. In Boston, 13 of 44 (30%) mothers had less than a high school diploma, 23 (52%) had finished high school only. Mother versus technician paired swabs (*n* = 62) had similar pathogen yield (paired t-test and Wilcoxon signed rank test *p*-values = 0.62 and 0.63, respectively; 95% confidence interval of the difference between the two measurements = − 0.45–0.75). Median Ct value for RNP was 22.6 (interquartile range, IQR = 2.04) for mother-collected and 22.4 (IQR = 2.39) for technician-collected swabs (*p* = 0.62). Agreement on pathogen yield between samples collected by mothers vs. technicians was higher for viruses than for bacterial pathogens, with high concordance for rhinovirus/enterovirus, human metapneumovirus, and adenovirus (Cohen’s kappa coefficients ≥80%, *p* < 0.0001). For bacterial pathogens, concordance was lower to moderate, except for *Chlamydia pneumoniae*, for which kappa coefficient indicated perfect agreement.

**Conclusion:**

Mothers with a range of education levels from low-income communities were able to swab their children equally well as technicians. Home-swabbing using dry tubes, and less invasive collection procedures, could enhance respiratory disease surveillance.

**Supplementary Information:**

The online version contains supplementary material available at 10.1186/s12889-022-12523-3.

## Background

Specimens collected from the upper-respiratory tract can be used to help guide patient management and infection control measures and are widely used as a surveillance tool to monitor circulation of respiratory pathogens [1–3]. Home-based swabbing to collect respiratory samples has not been widely used but could support control measures to reduce transmission levels in the community such as isolation of cases, mask wearing and interventions like contact tracing, and to enhance patient access to testing, reducing the pressure on healthcare systems during public health crises [4, 5]. During the 2009 influenza pandemic, for example, England was able to better ascertain disease incidence in communities by relying on self-swabbing and shipment of samples to reference laboratories [6]. With the COVID-19 pandemic, some recent studies have explored the reliability of home-based swabbing, specimen quality, and strategies related to choices in specimen types and transport media [7–10]. Nonetheless, performance data of home-based swabbing among underserved communities are limited, especially in areas where lack of health insurance and language barriers might reduce access to community health facilities, accentuating inequities in morbidity [11]. In our study of home-based swabbing among low-income populations in two U.S. cities, we asked mothers to collect respiratory swabs from their children and compared those with swabs collected by technicians.

## Methods

This retrospective data analysis was drawn from the Green Housing Study, which was designed to explore health outcomes among families with children ages 7–12 years with asthma who lived in low-income federal government-subsidized housing. The study focused on two low-income communities—one in Cincinnati, Ohio, which was entirely African American, and the other in Boston, Massachusetts, which was predominantly Chinese and Latino. One child per household was selected for this study [12, 13].

We asked mothers to collect nasal and throat swabs from their child when the child experienced an episode of acute respiratory illness (ARI). ARI was defined as the presence of three or more of the following symptoms for > 24 h: fever, stuffy/runny nose, cough, sore throat, body aches, or fatigue, or whenever the child was thought to have a cold or “the flu”.

We gave each mother a sample collection kit with storage and handling instructions (See Supplementary for instruction leaflet). To ensure that the mother understood the procedure, the study coordinator offered to help swab the child during the enrollment visit. Mothers were told to contact the study coordinator for specimen pickup as soon as they swabbed their child. We analyzed the ability of mothers to collect swabs from their child by comparing pathogen yield and quality of specimens with those obtained by trained technicians.

### Specimen collection

Participating mothers were advised to collect respiratory specimens within 24–36 h of their child’s symptom onset. We asked parents to store samples in their refrigerator until pickup by the technician as close in time as possible to when the mother swabbed the child (but not ≥5 days after symptom onset). During the pickup visit, the technician also collected nasal and throat swabs from the child using the same instructions provided to the mothers. Specimens were collected using a pair of flocked swabs. One swab was placed into each nostril and a second one was used to swab the posterior pharynx. Nasal and throat swabs were combined in a dry tube. A set of controls was introduced mid-study, we asked mothers and technicians to swab asymptomatic children on pre-defined days. All swabs were transported on ice to the reference laboratory and stored at − 70 °C until testing at the Division of Viral Diseases laboratory, Centers for Disease Control and Prevention (CDC), Atlanta, GA. Samples were analyzed in batches within the study period (2011–2013).

### Laboratory testing

Specimens were tested using TaqMan array cards, a microfluidic, 384-well real-time PCR platform capable of rapid, simultaneous testing of multiple clinical specimens for a variety of respiratory pathogens (including viruses and bacteria), plus controls [14]. The cards also feature a human specimen control assay that detects the ribonuclease P (RNP) gene we used as a proxy for the quality of samples collected by mothers vs. technicians. Cycle threshold (Ct) values are inversely related to the copy number of human or viral RNA; Ct < 40 was interpreted as a positive result.

### Analysis

We assessed concordance and discordance of detectable pathogens from mother-collected nasal and throat swabs compared with those collected by technicians using Cohen’s Kappa coefficients [15]. We applied Landis and Koch guidelines for interpretation of kappa values where 0.41 to 0.60 indicates moderate agreement, 0.61 to 0.80 indicates substantial agreement, and 0.81 to 1.0 almost perfect agreement) [16]. Boxplots were created and Pearson’s correlation coefficients calculated to compare RNP Ct values from mother-collected vs. technician-collected samples. Parametric paired t-tests and non-parametric Wilcoxon signed-rank test due to non-normal distribution and the small sample size were used to examine statistically significant differences between the two measurements. We also examined concordance of pathogen detection and quality of specimen comparing mother-collected vs. technician-collected swabs stratified by whether the child was swabbed while symptomatic or as asymptomatic (i.e., part of the set of controls). Bland-Altman plots were performed to also show degree of agreement between the RNP Ct values of the paired samples comparing mother vs. technician collected specimens and to contrast findings with that from correlation coefficient [17].

### Ethical considerations

Bilingual technicians in Cantonese and Mandarin or fluent in Spanish explained the study procedures to the participants. The technicians obtained informed consent from mothers and assent from children. The study was approved by Institutional Review Boards of CDC and the Harvard T.H. Chan School of Public Health.

## Results

During 2011–2013, we enrolled 80 households in the study: 36 in Cincinnati and 44 in Boston. All 36 Cincinnati households identified their children as Black or African American. Among the 44 mothers in households in Boston, 37 (84%) were born outside the U.S. mainland. These included 27 (61%) born in China, with Cantonese or Mandarin as their spoken language, and 12 (26%) reported their children were of Hispanic/Latino ethnicity. In Cincinnati, eight of 32 (25%) mothers did not finish high school, 11 (34%) had finished high school only, and 13 (41%) had enrolled in college. In Boston, 13 of 44 (30%) mothers had less than a high school diploma, 23 (52%) had finished high school only, and eight had enrolled in college. Among mothers in Cincinnati, 19 of 32 (59%) reported smoking cigarettes, compared with one of 43 (2%) in Boston (Table 1).

**Table 1:** Characteristics of Enrolled Households and Participating Children in Cincinnati, Ohio, and Boston, Massachusetts, USA

Characteristic	Cincinnati n/N (%)	Boston n/N (%)
Sex of child		
Male	17/36 (47)	25/44 (57)
Female	19/36 (53)	19/44 (43)
Race of child		
White	0/36 (0)	9/44 (20)
Black or African American	36/36 (100)	4/44 (9)
Asian	0/36 (0)	27/44 (61)
Native Hawaiian or Other Pacific Islander	0/36 (0)	0/44 (0)
American Indian or Alaska Native	0/36 (0)	0/44 (0)
White and Asian	0/36 (0)	1/44 (2)
Other	0/36 (0)	3/44 (7)
Ethnicity (Hispanic/Latino) of child	0/36 (0)	12/44 (26)
Overall characteristic of households ^a^		
Number of children living in the household		
1	6/31 (19)	8/44 (18)
2	7/31 (23)	21/44 (48)
3	10/31 (32)	13/44 (30)
4	4/31 (13)	1/44 (2)
5	3/31 (10)	0/44 (0)
6	1/31 (3)	1/44 (2)
Mother reported smoking cigarettes		
No	13/32 (41)	42/43 (98)
Yes	19/32 (59)	1/43 (2)
Language spoken at home		
English and Spanish, but mostly English		3/44 (7)
English and Spanish, but mostly Spanish		4/44 (9)
Only English	32/32 (100)	5/44 (11)
Only Spanish		2/44 (5)
Other		30^b^/44 (68)
Mother’s birthplace		
Mainland USA	32/32 (100)	7/44 (16)
Puerto Rico		2/44 (5)
Other		35^c^/44 (80)
Mother’s highest education level		
Less than high school diploma, no GED	8/32 (25)	13/44 (30)
High school diploma or GED	11/32 (34)	23/44 (52)
Some college but no degree	11/32 (34)	2/44 (5)
Associate degree	2/32 (6)	5/44 (11)
Bachelor’s degree (e.g., BA or BS)		1/44 (2)
Mother is employed outside of the home		
No	24/32 (75)	21/43 (49)
Yes	8/32 (25)	22/43 (51)
During the past 3 months (from enrollment into study), child had an episode of asthma or an asthma attack?	13/36 (36)	17/44 (39)
During asthma attack reported in the past 3 months, child visited emergency department	7/13 (54)	5/13 (28)
Child vaccinated against influenza during past year	19/33 (58)	31/37 (84)

*Abbreviations*: *GED* General Educational Diploma, *BA* Bachelor of Arts, *BS* Bachelor of Science

^a^Denominators vary by question because of incomplete reporting by mothers

^b^These included 28 Cantonese or Mandarin and two unspecified

^c^These included 27 from China, five from the Dominican Republic, one from Niger, one from Germany, and one from Haiti

In both cities, mothers reported similar rates (approximately 36%) of a child having at least one episode of asthma attack in the previous 3 months from time of enrollment. During that period, seven of 13 (54%) children in Cincinnati visited an emergency department for asthma attack, compared with five of 13 (28%) in Boston. Among the children in Cincinnati, 19 of 33 (58%) had received influenza vaccinations, as had 31 of 37 (84%) of the children in Boston (Table 1).

A total of 190 swabs were collected from December 2011 through November 2013. Those included 62 mother-technician pairs of nasal and throat swabs (*n* = 124 swabs) collected from symptomatic (*n* = 44 swabs) and asymptomatic (*n* = 80 swabs) children. The other 66 swabs were collected only by mothers, either from asymptomatic children during the enrollment visit (*n* = 40) or when their child was ill, but a technician was not able to collect a paired sample on time (*n* = 26). Of the 62 paired samples, we had complete date of collection for 53 pairs. The median difference in days between parental vs. technician swabbing was 1 day (range: 0–5 days).

Agreement on pathogen yield between samples collected by mothers vs. technicians was higher for viruses than for bacterial pathogens, with high concordance for rhinovirus/enterovirus, human metapneumovirus, and adenovirus (Cohen’s kappa coefficients ≥80%, *p* < 0.0001). For bacterial pathogens, concordance was lower to moderate, except for *Chlamydia pneumoniae*, for which kappa coefficients indicated perfect agreement (Table 2). Similar results were observed when the analysis was performed separately for asymptomatic and symptomatic children using paired swabs (data not shown).

**Table 2:** Concordance/discordance of mother-collected swabs vs. technician-collected paired swabs (*n* = 62 pairs) from households in Cincinnati, Ohio, and Boston, Massachusetts, USA^a^

Pathogen^**b**^	Detected in either Mother or Technician collected swabs	Concordant pairs		Discordant pairs		Kappa coefficient (95% CI)
		Mother negative and Technician negative (−/−)	Mother positive and Technician positive (+/+)	Mother negative and Technician positive (−/+)	Mother positive and Technician negative (+/−)	
**Any viruses**	21	40	15	3	3	0.76** (0.58–0.94)
Rhinovirus/Enterovirus	16	46	11	2	3	0.76** (0.57–0.96)
Influenza A	1	61	1	0	0	1.0** (1.0–1.0)
Influenza B	1	61	1	0	0	1.0** (1.0–1.0)
Human Metapneumovirus	2	60	2	0	0	1.0** (1.0–1.0)
Adenovirus	1	61	1	0	0	1.0** (1.0–1.0)
Human Coronavirus OC43	2	60	1	1	0	0.66** (0.04–1.0)
Parainfluenza 3	2	60	0	1	1	−0.02 (− 0.04–0.01)
**Any bacteria**	54	7	40	9	5	0.36 (0.08–0.62)
*Haemophilus influenzae*	25	37	12	6	7	0.50** (0.26–0.73)
*Streptococcus pneumoniae*	27	34	14	7	7	0.50** (0.27–0.72)
*Chlamydia pneumoniae*	2	60	2	0	0	1.0** (1.0–1.0)
*Group A Streptococcus*	10	52	3	4	3	0.40* (0.04–0.76)
*Moraxella catarrhalis*	14	48	9	1	4	0.73** (0.52–0.95)
*Staphylococcus aureus*	39	22	25	9	5	0.52** (0.30–0.73)

*Abbreviations*: *PPV* positive predictive value, *NPV* negative predictive value

* *p* < 0.01, ** *p* < 0.0001

^a^Pathogens included in the TaqMan array card used in the study. Cycle threshold (Ct) < 40 was interpreted as a positive result

^b^The following pathogens were not detected in any of the samples: Influenza C, Human Coronavirus (229, NL63, and HKU1), Parechovirus, Parainfluenza 1, 2, and 4, Respiratory Syncytial Virus, *Legionella pneumophila, Bordetella pertussis, and Mycoplasma pneumoniae*

**Fig. 1:**
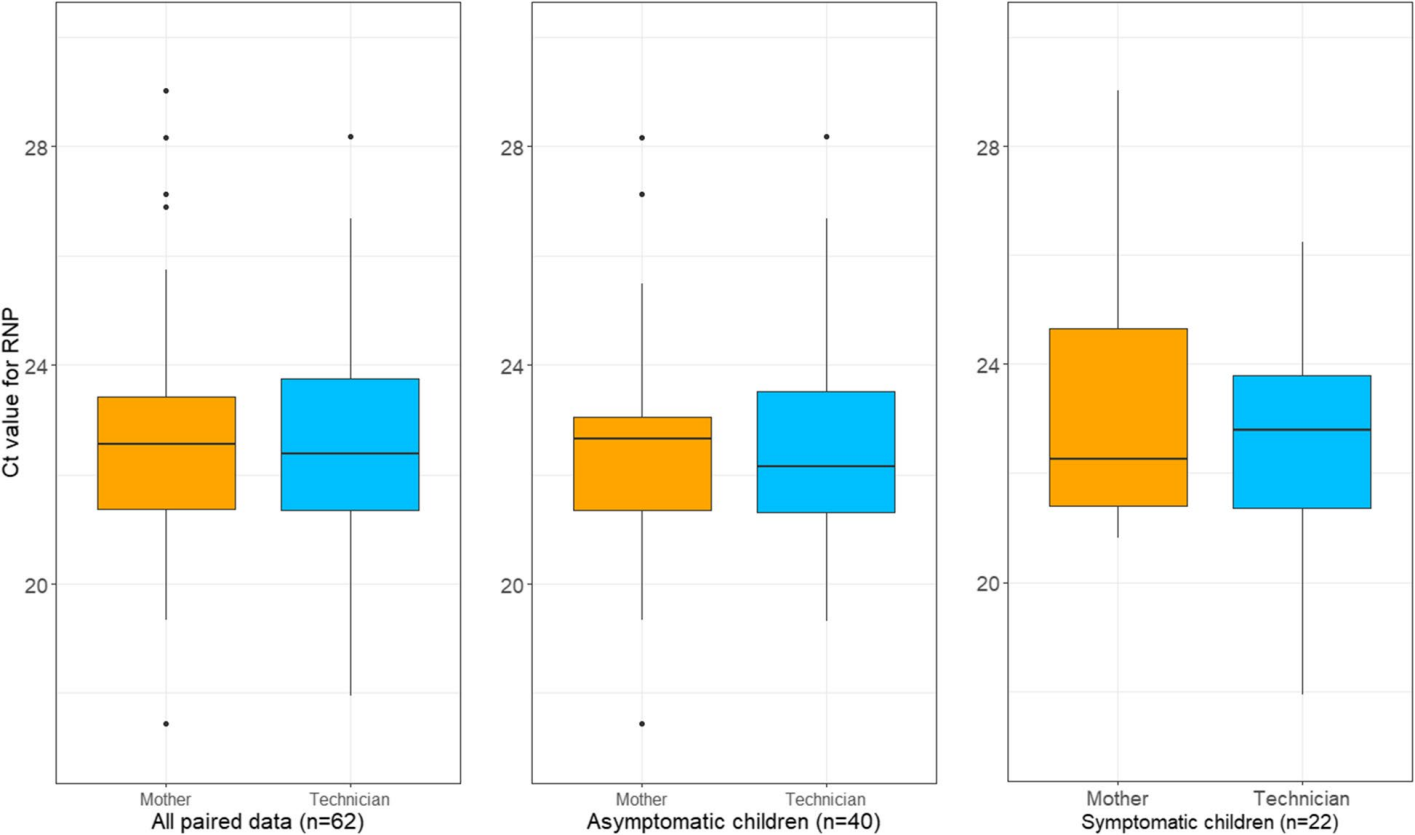
Boxplots in panel (**A**) display the distribution of cycle threshold (Ct) value for human ribonuclease P (RNP), comparing the quality of specimens collected by mothers with that collected by technicians (62 paired swabs). A paired t-test showed no significant difference in RNP value for the 62 swab pairs (mean of the differences = 0.15; *p* = 0.62). Panel **B** in the middle shows results from paired swabs collected from asymptomatic children (*n* = 40), *p* = 0.95; panel **C** on the right shows results from paired swabs collected from symptomatic children (*n* = 22), *p* = 0.39. The vertical line through each box shows the median. The whiskers go from each quartile to the minimum or maximum values

The median Ct values for RNP were 22.6 (IQR: 2.04) for the 62 mother-collected swabs and 22.4 (IQR: 2.39) for the paired technician-collected swabs (Fig. 1, panel A). A paired t-test showed no significant difference in RNP value for the 62 swab pairs (mean of the differences = 0.15; paired t-test and Wilcoxon signed rank test *p*-values = 0.62 and 0.63, respectively; 95% confidence interval [CI] of the difference between the two measurements was-0.45–0.75). The RNP values between the two were significantly correlated (*r* = 0.31; *p* = 0.01). When paired swabs were stratified by asymptomatic and symptomatic status (Fig. 1 – panel B and C), RNP values were still not significantly different between those collected by mothers vs. technicians (asymptomatic *p* = 0.95 [mean difference = − 0.02; 95% CI = -0.75–0.71]; symptomatic *p* = 0.39 [mean difference = 0.46; 95% CI = -0.64–1.57]). Non-parametric Wilcoxon signed-rank test *p*-values (0.92 for the asymptomatic and 0.59 for the symptomatic) were similar to those of the parametric paired t-test. The median Ct value for RNP from swabs collected by mothers (*n* = 66), not paired with technician-collected swabs, was 22.1 (IQR: 2.1). A Bland-Altman plot of the difference between the RNP values obtained from mother vs. technician collected samples against their mean was done, also considering asymptomatic and symptomatic pairs, validated the findings from correlation coefficient. See Supplementary Figure for plots.

## Discussion

With simple instructions and less invasive specimen collection procedures (i.e., anterior nasal swabs combined with oral swabs instead of nasopharyngeal swabs), mothers with predominately low education levels from low-income communities were able to swab their children equally as well as trained technicians. Moreover, the use of a dry tube, with no viral transport media, did not affect the viability of the specimens. RNP human specimen control Ct values and the yield of pathogens were comparable between samples collected by mothers and technicians. Our study demonstrates the utility of home-swabbing to ascertain disease among communities with less access to health facilities to improve overall understanding of respiratory disease patterns.

We demonstrate strong proficiency in home-based swab sample collection among mothers with lower levels of education and language barriers. An earlier pilot study focused primarily on an immigrant Latino population in New York City and reported good results for self-swabbing proficiency [18]. A similar study in Australia showed the yield of viral detections from parent-collected nasal and throat swabs from children to be comparable to healthcare worker-collected swabs. However, in contrast with mothers in our study, participating parents in that study had a higher education level, and most were healthcare professionals themselves [19].

Although nasal swabs might be less sensitive in detecting some viruses when compared with nasopharyngeal swabs or aspirates, the ease of anterior nasal collection procedures allows for self-collection and home-based collection of specimens [20]. Combining nasal and throat swabs improves pathogen yield and is less invasive, and therefore, more tolerable [10, 21]. Molecular methods based on reverse transcription-polymerase chain reaction are becoming increasingly available for identification of respiratory etiologies as part of routine care and surveillance activities. Because these tests are more sensitive than culture or antigen tests for many of the circulating pathogens [22], less invasive specimen collection with comparable yields becomes acceptable. Multipathogen molecular platforms like the one used in our study, detecting various respiratory pathogens simultaneously, can vary in sensitivity and specificity, which ultimately would need to be assessed when interpreting pathogen yield [14, 23–25]. However, our finding that mothers can collect respiratory specimens given simple instructions corroborates the use of home-swabbing in the context of public health response and surveillance initiatives.

To make samples easier to handle, we used dry tubes. This did not affect the quality of the samples collected by mothers and technicians, as measured by RNP Ct values, which were 22 for both groups. We showed that respiratory swab specimens transported in dry tubes are stable up to 5 days if kept refrigerated (~ 4 °C). A recent SARS-COV-2 study validated the use of dry polyester and foam surrogate swabs by demonstrating that samples were stable through 72 h when refrigerated or after exposure to high temperature (simulating transport without cold chain) [8]. For other respiratory viruses, previous studies have confirmed good recovery of viral RNA from dry cotton or flocked swabs after storage for up to 2 weeks at room temperature [26]. Influenza virus detection rates from dry swabs stored for up to 5 days at room temperature in the same study were higher than those from paired samples collected in viral transport media and assayed by cell culture and immunofluorescence [26]. To improve monitoring of circulating respiratory viruses of public health importance, dry swabs should be considered for respiratory disease surveillance in areas with limited cold-chain access. Using dry swabs might also offset transport media shortages in times of high demand for respiratory testing (e.g., epidemics) and might be less costly than transport media, which tend to have limited shelf lives.

### Limitations

For this study, we were not able to confirm whether the yield of pathogens detected in samples stored in dry tubes would be the same as that stored in transport media because both groups used dry tubes for collection. Additionally, discussion of pathogen yields is only relative as standard curves were not used to perform absolute quantitation. Dry storage might not be adequate for influenza surveillance as performed by some national influenza centers that need to isolate viruses for vaccine composition. We also were not able to explain the higher concordance between mothers and technicians in virus detection compared with bacterial pathogen detection. Perhaps, in the absence of an active infection, different timing of swabbing in the same site could yield slightly different results for a low titer target. This is something to further explore in studies of bacterial colonization of the upper respiratory tract.

## Conclusions

The ability to use home-based anterior nasal and oral swabbing, combined with storage and shipment of dry clinical specimens for virus testing, could significantly improve respiratory disease surveillance in remote or resource-limited settings, and could reduce the demand on the healthcare system in times of public health crisis. Further validation of this approach at large scale should be considered, and discussions on the relevance of this approach to improve respiratory pathogen surveillance in the U.S. are warranted.

## Supplementary Information

**Additional file 1.**

## Data Availability

The datasets used and/or analyzed during the current study are available from the corresponding author on reasonable request.
