# Supplementary material for: Side-by-side comparison of parent vs. technician-collected respiratory swabs in low-income, multilingual, urban communities in the United States
Source: BMC Public Health. 2022 Jan 15;22:103. doi: 10.1186/s12889-022-12523-3 (PMC8760092; doi:10.1186/s12889-022-12523-3)
Supplement: Supplementary file 1 — Additional file 1. [file 12889_2022_12523_MOESM1_ESM.docx]

**Supplementaty – Instruction leaflet given to mothers during enrollment**


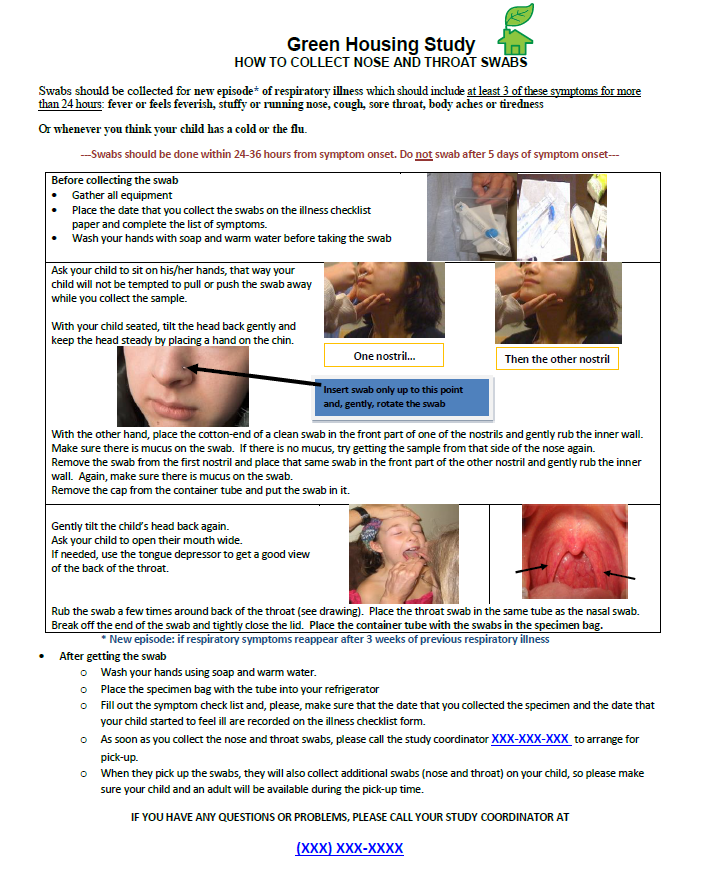


**Supplementary - Figure.**


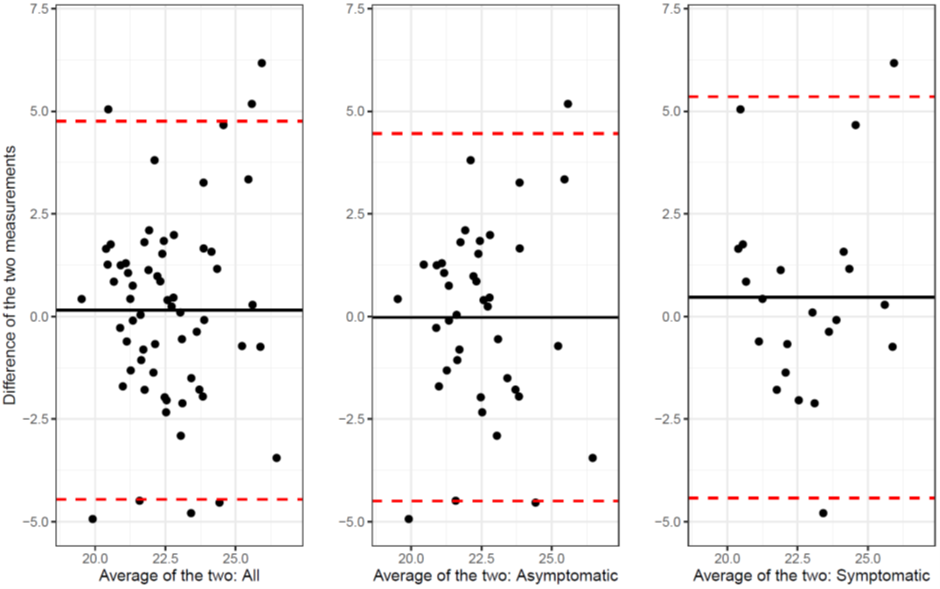


Bland-Altman plot for cycle threshold (Ct) value for human ribonuclease P (RNP), comparing the quality of specimens collected by mothers with that collected by technicians (62 paired swabs). Panel A on the left shows Bland Altman plot for all data; panel B in the middle shows results from paired swabs collected from asymptomatic children; and panel C on the right shows results from paired swabs collected from symptomatic children. The black real lines represent average value of the differences between Ct values of specimens collected by mothers and technicians. The red dashed lines represent 95% confidence interval of the differences.
